# Rev. Mr. Coleridge's History of Small-Pox a Second Time

**Published:** 1817-01

**Authors:** W. H. Coleridge

**Affiliations:** St. Andrew's-court, Holborn


*For the London Medical and Physical Journal.*

[Dr. Adams has favoured us with the following history of a second small-pox, all the symptoms being related by the subject himself, the Rev. Mr. Coleridge, Minister of St. Andrew's, Holborn.]

MY DEAR SIR,

IN 1789, a woman, with a child in her arms extremely ill of the small-pox, which was then very general in the village, came to consult my father. By mistake she brought the child into the room in which I was, and remained a considerable time at the door, till my father arrived. This child had the confluent small-pox, and died on the following day; and the woman, who had probably taken the infection from the child, had it very badly, but recovered. I was then about ten days old. My father concluded that I should be infected, but concealed his fears from my mother, who some time after perceived that I was unwell, appeared dull and heavy, and made a constant moaning. Shortly after some eruptions appeared in various parts of my body, to the number of fifteen: these came to maturity, and fell off in the usual way; and it was not till then that my father informed my mother that I had had the small-pox in the most favourable manner, and related the way in which he supposed that I had received the infection. As my father was a medical man, and in the constant habit of seeing the small-pox, it is not probable that he should have been under a mistake. To this I would add the testimony of my nurse, and another medical person, who saw me whilst under the disease; and my frequent exposure to infection since that time without any ill consequences, especially at the age of three years, when when I played and mixed with a family of children during the whole time they were under the small-pox. The fact, therefore, of my having had the small-pox in my infancy seems to be placed beyond all reasonable doubt.

On the 26th of October, 1816, I was sent for to baptize a child in King's Head-court, Gray's Inn-lane. On my arrival, I found the family in a small room on the first floor, as the end of the alley. In one corner was a coffin, in which was the body of a child that had died of the small-pox. On the knee of a woman was another, very ill in the confluent sort; and on the mother's lap a third, on which the eruptions were just appearing. The window was closed, to prevent, as they said, the dead body from turning too soon; and, from the closeness of the room, and the unpleasant smell, it had probably not been opened for some days. After I had baptized the child, I remained some time longer, conversing with the parents, but did not then experience any peculiarly unpleasant sensations from the state of the air.

On the morrow I called again. On my entrance the smell was much more disagreeable than before; and, on my opening my mouth to speak, such effluvia passed down my throat as almost turned my stomach. This visit did not last more than a few minutes; and, as I had taken the usual precaution of not swallowing the saliva, and had already had the small-pox, I felt no alarm from this circumstance.

From that day to the 9th of November, I enjoyed very good health. In the afternoon I was exposed for some time to a draft in rather a warm room, and, feeling myself very weary and listless in the evening, I concluded that I had taken cold, and, in consequence, retired early to bed. On the following day, which was Sunday, I could take but little care of myself. I felt qualmish, my head was unsteady, and I had a dull aching pain in my legs. Some magnesia, however, gave me temporary relief, and enabled me to go through the duties of the day. On the Monday these symptoms returned, though the unsteadiness which I had felt was exchanged for a dull bursting pain in my forehead, and particularly between the eyes, which were now so weak that I could not bear the light. My appetite was gone; my pulse were quick; my tongue coated. From these I was again partially relieved by some more magnesia and rhubarb. I should have mentioned that I had, now and then, for a few moments, a passing pain in my back; but I do not remember feeling any pain in the pit of the stomach. On Tuesday I found myself so unwell, that I thought it proper to call in medical aid. The medicines prescribed for that and the following evening threw me each time into a violent perspiration, spiration, and acted rather powerfully on the bowels. After two or three hours of unrefreshing sleep, on the Wednesday night, I awoke about midnight, and lay for five hours unable to sleep. My mind passed in rapid succession from one idea to another, without my having the power to fix it for a moment almost on any one; and my feelings in my head were such, that I concluded I was going to have nothing less than a brain fever. This long wakefulness was succeeded by a short sleep, from which I was soon roused by a highly delirious dream. My next slumber was more composed, and I felt refreshed; and, to my great astonishment, I arose at noon, and felt as if nothing more was the matter with me than that I was unusually weak. I was surprised, however, to perceive my forehead, and several parts of my body, covered with eruptions. On the Friday they were more numerous and distinct; and it was not till then that any suspicion of my real complaint arose, or the occurrence of the 27th was brought to my mind, by a question whether I had ever been vaccinated, or had had the small-pox. The relation of this strengthened the suspicion, and the appearance of the eruption on the Sunday put the matter beyond all doubt. The pustules have gone through the usual stages, and I am now almost entirely recovered.

I must apologize, &c. [the remainder of the letter is on a different subject], and am, my dear Sir,

Your most obedient and humble Servant,

*St. Andrew's-court, Holborn;* W. H. Coleridge.

*Dec.* 7, 1816.

P.S.—Should you wish to make any further enquiries, it may be proper to mention that I was attended by Dr. Southey and Mr. Wells. Dr. Southey, however, was called away on the Tuesday night, and did not return till it was decided that I had the small-pox.

*(To Dr. Adams, Hatton Garden.)*

